# Lifestyle, nutritional, and health influences on consumption of artificially-sweetened beverages in educated urban populations

**DOI:** 10.1371/journal.pone.0322553

**Published:** 2025-04-29

**Authors:** Nutnicha Loyfah, Aphichat Chamratrithirong, Rossarin Soottipong Gray, Umaporn Pattaravanich, Sasinee Thapsuwan, Natjera Thongcharoenchupong, Sirinya Phulkerd

**Affiliations:** Institute for Population and Social Research, Mahidol University, Nakhon Pathom, Thailand; Qassim University, SAUDI ARABIA

## Abstract

**Objectives:**

This study analysed the prevalence of artificially-sweetened beverages (ASB) consumption in the Thai population, and examined associations between ASB consumption and lifestyle, and nutritional and health-related behaviours.

**Study Design:**

This study used cross-sectional design using nationally-representative data from the 2021 Health Behavior of Population Survey.

**Methods:**

The study population was 8,155 Thai adults (age 20–64 years) who lived in an urban area and attained at least a bachelor degree education at the time of interview. Binary logistic regression was applied to investigate associations between ASB consumption and sociodemographic characteristics, lifestyle behaviors, and obesity in the study adults.

**Results:**

The study found that participants who were overweight/obese Class 1 had 1.5 times (OR = 1.49, 95% CI 1.18–1.88) and 1.4 times (OR = 1.36, 95% CI 1.08–1.72), respectively, more likely to ASB consumption than those with normal weight. Non-smokers and those who engaged in regular physical activity were more likely to have higher ASB consumption. Participants living with one or more non-communicable disease (NCD) were more likely to be a consumer of ASB than those without a NCD (OR = 1.56, 95% CI 1.24–1.97). Some socio-demographic characteristics (e.g., sex, age, geographic area of residence, education) were also associated with ASB consumption.

**Conclusions:**

The results suggest that government needs to develop effective communication strategies which convey accurate, clear, and concise information to consumers regarding the health effects of ASB consumption. This advice should be incorporated with interventions to improve food literacy, and in marketing control to improve individual consumption behavior and promote a healthier food environment.

## Introduction

In May 2023 the World Health Organization (WHO) launched new guidelines on non-sugar sweeteners (NSS), which include acesulfame K, aspartame, advantame, cyclamates, neotame, saccharin, sucralose, stevia, and other stevia derivatives [1]. The WHO recommendations point out that NSS may have undesirable health effects which include increased risks of disease, such as diabetes and cardiovascular illness. There may also be other adverse health effects such as the increased risk of premature death among adults. In addition to discouraging use of NSS, the WHO recommendations also warn against the use of all synthetic and naturally-occurring or modified non-nutritive sweeteners in manufactured foods and beverages[1].

Increased consumer health awareness and changing lifestyle toward healthy diets (including consumption of low-sugar and energy-dense foods and beverages) has been observed in recent years [2]. The NSS have become ubiquitous as common replacements for added sugar for reducing calorie content from free sugar, in general population and among specific group with obesity and diet-related noncommunicable diseases (NCD). They are being used widespread in global food supply, and are expected to grow up to 7.2% from 2022 to 2032, with an expected market value of US$ 46 Billion by the end of 2032[3].

Despite increasing evidence of the undesirable health impact of NSS, consumer perception of its safety (or hazard) remains low. According to a US adult consumer survey, almost one-third consumers perceived that non-nutritive sweeteners can be part of their overall healthful diet, while 73% of those consuming theses sweeteners consumed them because they wanted to reduce total calories ingested [4]. More than one-third US consumers (36%) were not concerned about the safety of artificial sweeteners [5]. As for urban growth, a wide use of artificial sweeteners was observed in urban population. Use of saccharin, acesulfame and sucralose were reported in all urban canal water population in Hanoi, Vietnam [6]. In India more than one-third of urban populations consumed artificial sweeteners each month, which some reported consuming these sweeteners in more than one food product [7]. The UK survey of a working-age sample found that approximately 30% of participants considered non-nutritive sweeteners as harmful, 40% were concerned about their effects on human body and almost 30% were concerned about increase in cancer risk[8]. These concerns could be influenced by consumer knowledge, and related government policy and regulation that can affect perceived benefits and risks of consumption of sweeteners in the population [8–10].

A significantly increased consumption of artificially sweetened beverages (ASB) has been observed in recent years [11]. ASB intake appears to increase with age and is more common among girls than boys, and among women than men, and that people trying to lose weight report a higher intake of ASB than those not trying to lose weight [12,13]. In addition, ASB consumers are more likely to be college educated [14]. It is reasonable to assume that the observed increased trend in frequency of consumption of ASB is partly due to greater accessibility and selection of ASB in addition to the promotion of ASB as a healthier alternative to SSB [15]. A study on consumption of sugar-sweetened beverages (SSB) and ASB from childhood to adulthood in relation to socioeconomic status – 15 years follow-up in Norway found a decrease in SSB consumption and an increase in ASB consumption from childhood to adulthood [16].

There are growing concerns about safety of NSS consumption in Thailand. Nonetheless, the published research on this topic includes only a limited number of small observational studies on ASB consumption in Thais [17,18]. Furthermore, none of those studies paid attention on the impacts of the rise of urbanisation on Thais’ diets and influence of education attainment on perception on ASB. No studies have used nationally representative data, explored key characteristics of Thais consuming ASB, or examined associations between ASB consumption and influential factors. Therefore, this study had the following two objectives: (1) To document the prevalence of ASB consumption in educated Thai adults in urban areas; and (2) To examine associations of ASB consumption with sociodemographic characteristics, lifestyle behaviors, and obesity in the study adults. The source of data is a large nationally-representative sample of the Thai population. Findings of this study should be useful for informing policy decisions and communication strategies to combat the harmful effects of NSS consumption in Thailand.

## Methods

### Study design and participants

This study used data from the 2021 population-based survey called the Health Behavior of Population Survey (HBPS). The HBPS was the largest population-based survey on NCD and their risk factors in Thailand conducted by the National Statistical Office of Thailand (NSO) [17]. The HBPS contains data from a nationally-representative sample, covering information on sociodemographic characteristics and food consumption of the Thai population age six years or over. Data collection was conducted during February – May, 2021.

The HBPS employed a two-stage sampling procedure to recruit study participants. Across all the major geographic regions, a systematic sampling of Thailand’s 77 provinces was conducted. From these selections, 20 enumeration areas (EA) within each province were systematically sampled. Within each EA, 16 households were chosen, and subsequently contacted. A list of family households was obtained. All household members who were eligible were invited to participate in the survey. However, it should be noted that the level of data accuracy from this survey can be affected by the sampling method. The NSO reported the percentage of coefficient of variation of main characteristics such as between consuming sugar-sweetened beverages, and age, sex and area of residence (rural, urban) ranging from 0.90–2.84 [17] which can be regarded as the excellent level of data accuracy.

The protocol for this study was initially reviewed by the Institutional Review Board of Institute for Population and Social Research of Mahidol University (COE. No. 2023/03–044). However, it was then exempt from further review for the current secondary analysis because it did not meet the criteria for human subjects’ research.

The NSO research team contacted the sampled households through a village leader in each EA. The interviews took approximately 30–45 minutes each. Respondents were given an opportunity to ask for clarification. A total of, 84,000 members of sampled households were successfully interviewed face-to-face by trained staff using a structured questionnaire.

There was a total of 62,891 eligible cases, as shown in S1 Table. In order to reach the prioritized target population, this study focused specifically on Thais age 20–64 years, living in an urban area, and had attained at least a Bachelor’s degree. There is evidence elsewhere that this population group had high SSB consumption [19], and are more likely to access/consume NSS compared with other population groups [20, 21]. That filter produced a net sample of 8,155 for this study.

### Measures

#### Dependent variable.

Participants were asked about frequency of ASB consumption using the following question “In the past 30 days, how often did you consume an artificially sweetened beverage (ASB)?” Participants who reported consuming ASB at least once per week, which include consuming 1–2 days, 3–4 days, 5–6 days, or every day, were classified as “consume.” Those who did not consume ASB at all or consumed it only 1–3 days per month were classified as “not consumed”.

#### Independent variables.

Socio-demographic characteristics: Sex was included as a dichotomous variable, i.e., “*male*” or “*female*.” Age was categorised into two groups: “*20-49 years*”, and “*50-64 years*.” Marital status was classified into “*single*,” “*married*,” and “*widowed/divorced/separated*.”

Socio-economic status: For monthly cash income, participants who earned “less than or equal to 2,803 baht” and “*more than 2,804 baht*” were classified as being “*poor*” and “*not poor,*” respectively, according to Office of the National Economics and Social Development Council. Geographic area of residence was categorised into five groups: “*Bangkok*,” “*Central*,” “*North*,” “*Northeast*,” “*South*.”

Lifestyle behaviours: Smoking behavior was categorised into two groups: “*no*” (never smoke or used to smoke) and “*yes*” (smoke every day or smoke, but less than daily). Alcohol drinking was categorised into two groups: “*no*” (never drink, used to drink a year ago, drink 1–3 days per month, drink 8–11 days per year, drink 4–7 days per year, drink 1–3 days per year) and “*yes*” (drink every day, drink 5–6 days per week, drink 3–4 days per week, drink 1–2 days per week). Physical activity was categorised into two groups: “*no*” (not engaging in any vigorous exercise or playing sports) and “*yes*” (specifying the type of exercise or sport).

Health condition: Respondents were asked whether they currently have non-communicable disease(s). Response was divided into two groups: having at least one NCD -- classified as “*yes*”, and not having any NCD -- classified as “*no*.”

Nutritional status: Body Mass Index (BMI) was used as an indicator of overall nutrition status of the adult population. BMI was categorised into five groups: “*Underweight (BMI < 18.5),”* “*Normal weight (BMI 18.5–22.9),”* “*Overweight (BMI = 23.0 - 24.9),” “Obesity Class 1 (BMI = 25.0–29.9),” and “Obesity Class 2 (BMI ≥ 30)*,” respectively, according to WHO standards [22].

#### Statistical analysis.

Descriptive statistics were used to analyse the socio-demographic and-economic characteristics, lifestyle behaviours, health condition, nutritional status, and ASB consumption. Binary logistic regression was applied to investigate associations between ASB consumption and sociodemographic characteristics, lifestyle behaviors, and obesity. The associations are presented as odds ratios (OR) with 95% confidence intervals (with p-values). The p-values were derived using the Chi-squared (χ^2^) test. The threshold for statistical significance of associations was set at p < 0.05. This study used the survey weighted data, thereby ensuring a more precise representation of the target population.

## Results

### Population description

Overall, sample characteristics are presented in S1 Table. Many survey participants were female (56.8%), obtained primary school education or less (43.1%), were married (62.9%), lived in a rural area (51.9%), were not poor (82.4%), did not smoke (82.2%), did not drink alcohol (85.7%), engaged in regular physical activity (42.9%), and had a normal weight (43.9%). The majority of the participants did not consume ASB (96.7%).

#### Characteristics of participants age 20–64 years resided in an urban area and attained at least a Bachelor’s degree, and their ASB consumption.

Table 1 shows key characteristics of study participants (age 20–64 years, living in an urban area and attaining at least a bachelor’s degree) who consumed ASB. They were female (57.0%), participants were age 20–49 years (73.5%), were married (51.8%), were lived in a Bangkok (33.1%), were not poor (92.1%), did not smoke (89.5%), did not drink alcohol (89.2%), engaged in regular physical activity (59.3%), did not have NCD disease (85.1%), and had a normal weight (47.1%). The participants did consume ASB (6.6%).

**Table 1:** Socio-demographic and socio-economic characteristics of the ASB consumption among participants age 20-64 years, who resided in an urban area and attained at least a Bachelor’s degree (N = 8,155).

Variables	Number (Percent)		ASB Consumption (%)		χ^2^ (p-value)
			Yes	No	
Total	8,155	(100.0)	6.6	93.4	
Sex					P = 0.00
Male	3,509	(43.0)	5.8	94.2	
Female	4,646	(57.0)	7.3	92.7	
Age					P = 0.00
20-49	5,996	(73.5)	5.6	94.4	
50-64	2,159	(26.5)	9.6	90.4	
Marital status					P = 0.16
Single	3,294	(40.4)	6.0	94.0	
Married	4,224	(51.8)	7.1	92.9	
Widowed, divorced, separated	637	(7.8)	6.9	93.1	
Area					P = 0.00
Bangkok	2,669	(33.1)	7.7	92.3	
Central	2,470	(30.3)	8.0	92.0	
North	966	(11.8)	3.8	96.2	
Northeast	1,144	(14.0)	5.9	94.1	
South	875	(10.7)	3.4	96.6	
Poverty					P = 0.04
Poor	645	(7.9)	8.5	91.5	
Not Poor	7,510	(92.1)	6.5	93.5	
Smoke					P = 0.00
No	7,299	(89.5)	7.0	93.0	
Yes	856	(10.5)	3.3	96.7	
Drink alcohol					P = 0.16
No	7,272	(89.2)	6.8	93.2	
Yes	883	(10.8)	5.5	94.5	
Physical activity					P = 0.00
No	3,316	(40.7)	4.6	95.4	
Yes	4,839	(59.3)	8.1	91.9	
Disease					P = 0.00
No	6,938	(85.1)	5.9	94.1	
Yes	1,217	(14.9)	11.2	88.8	
BMI					P = 0.00
Underweight	488	(6.0)	4.5	95.5	
Normal weight	3,844	(47.1)	5.8	94.2	
Overweight	1,586	(19.4)	8.1	91.9	
Obesity Class 1	1,765	(21.6)	7.5	92.5	
Obesity Class 2	472	(5.8)	8.1	91.9	

No = not consumed in the past week; Yes = consumed in the past week

The participants who were female, age 50–64 years, were married, resided in the Central region, had healthy lifestyles (no smoking, no alcohol drinking, having physical activity), and had a NCD and were overweight/obese had a high percentage of ASB consumption compared with other variable(s) under the same category.

Significant relationships were observed between certain socio-demographic (e.g., sex, age, geographic areas of residence; p = 0.00) and socio-economic (e.g., poverty; p = 0.04) characteristics and ASB consumption among the study participants. The study also found relationships of ASB consumption with lifestyle behaviours (e.g., smoking, physical activity; p = 0.00), health condition (p = 0.00), and BMI (p = 0.00).

### Associations between BMI, health condition and socio-demographic and lifestyle characteristics, and ASB consumption

Results of the binary logistic regression analysis are presented in Table 2. There were statistically significant associations between BMI, some lifestyle behaviors and some socio-demographic characteristics, and ASB consumption among the study participants.

**Table 2:** Associations between study variables and ASB consumption among participants age 20-64 years, who resided in an urban area and obtained at least a Bachelor’s Degree (N = 8,155).

Variables	P-value	OR	95% CI for EXP(B)	
			Lower	Upper
Sex				
Male (Ref = Female)	.00	.73	.59	.89
Age				
20-49 (Ref = 50–64)	.00	.70	.57	.86
Marital Status (Ref = Widowed, divorced, separated)				
Single	.65	1.08	.76	1.54
Married	.36	1.17	.83	1.63
Area (Ref = South)				
Bangkok	.00	2.32	1.56	3.43
Central	.00	2.58	1.74	3.83
North	.82	1.05	.64	1.73
Northeast	.00	1.82	1.17	2.83
Poverty				
Poor (Ref = Not poor)	.77	1.04	.77	1.41
Smoke				
No (Ref = Yes)	.00	2.11	1.39	3.20
Drink Alcohol				
No (Ref = Yes)	.68	.93	.67	1.29
Physical Activity				
Yes (Ref = No)	.00	1.91	1.57	2.33
Disease				
Yes (Ref = No)	.00	1.56	1.24	1.97
BMI (Ref = Normal weight)				
Underweight	.33	.80	.50	1.26
Overweight	.00	1.49	1.18	1.88
Obesity Class 1	.00	1.36	1.08	1.72
Obesity Class 2	.08	1.38	.95	2.01
Constant	.00	.03		

Participants who were overweight/obese Class 1 had 1.5 times (OR = 1.49, 95% CI 1.18–1.88), and 1.4 times (OR = 1.36, 95% CI 1.08–1.72), respectively, more likely to consume ASB than those with normal weight.

The participants with no smoking (OR = 2.11, 95% CI 1.39–3.20), and having physical activity (OR = 1.91, 95% CI 1.57–2.33) were more likely to have higher ASB consumption than those who smoked and had no physical activity, respectively. Participants living with NCD more likely to consuming ASB than those living without an NCD (OR = 1.56, 95% CI 1.24–1.97).

Some socio-demographic characteristics (e.g., sex, age, geographic area of residence, education) were associated with ASB consumption. For example, males were 26.9% less likely to consume ASB (OR = 0.73, 95% CI 0.59–0.89) than females. Participants age 20–49 years had 29.4% less likely to consume ASB than those age 50–64 years (OR = 0.70, 95% CI 0.57–0.86). Participants who lived in Bangkok (OR = 2.32, 95% CI 1.56–3.43), the Central (OR = 2.58, 95% CI 1.74–3.38), and the Northeast region (OR = 1.82, 95% CI 1.17–2.83) were more likely to consume ASB than those in the South.

## Discussion

This study describes the prevalence of ASB consumption in a national sample of the Thai population who primarily lived in an urban area and were age 20–64 years and obtained at least Bachelor’s degree. To our knowledge, this is the first study of key characteristics of Thais who consumed ASB, and examined the relationships between lifestyles, and nutritional and health characteristics and ASB consumption. This study found significant differences in ASB consumption by all sociodemographic and economic, lifestyle, and health factors. Strong associations were observed among consumers with high BMI, living with an NCD and healthy lifestyle behaviours.

In this study, participants who were overweight/obese Class 1 were more likely to have high ASB consumption. The control of weight comes in various forms, with the fundamental principle being that those seeking weight loss must manage their food intake and modify behavior, including regular, vigorous physical exercise. Therefore, artificial sweeteners are often introduced as substitutes for sugar, and their usage has increased [23]. There is evidence that sweeteners replacing sugar have advantages but do not contribute to weight loss [24]. Instead, they may lead to weight gain that may be due to increased consumption of food, driven by the accustomed sweetness [25,26]. Ironically then, in murine studies, NSS have a more significant impact on weight gain compared to the consumption of sugar [27]. Individuals who are at higher risk for weight gain or have high BMI may misunderstand the effect of ASB consumption on nutritional outcome and, thus, choose to consume ASB in an attempt to control their weight or reduce BMI. Some food companies showed careful consideration in replacing sugar with artificial sweeteners due to consumers’ concerns on health effects [28]. Therefore, given perceived concerns about the health safety of artificial sweeteners, it is important for the Thai government to develop effective communication strategies which convey accurate, clear, and concise information to consumers regarding the potentially-adverse health effects of ASB consumption. It is also important for the government to monitor and control ASB marketing strategies, especially their messaging that must be cautious about explicitly mentioning the health effects of the ASB products.

A statistically-significant association between NCD and ASB consumption was observed in this study. Participants who have one or more NCD were more likely to have high ASB consumption. This is not surprising, and could be explained by the fact that people with disease may compensate for the energy deficit which refers to a lack of energy resulting from various health-related circumstances, which compel the body to find ways to compensate for the energy shortfall. This may include increasing food intake or opting for non-nutritive sweeteners to maintain an appropriate energy balance by replacing caloric SSB with ASB in order to reduce or control their health risks [8,29]. This phenomenon may also be the result of the global recommendation to reduce free sugar intake as part of a healthier diet, advocated by many organisations such as WHO. That recommendation entails reducing non-essential energy intake, excess body weight, and diet-related NCD in general [30]. Nevertheless, there is increasing evidence of the association between higher artificial sweetener consumption and increased health risks [31–33]. A large prospective cohort study in France found association between total artificial sweetener intake and increased cardiovascular diseases risk (95% CI: 1.01–1.18, P = 0.03) [31]. Another large cohort study in the US found associations between high ASB consumption and higher risks of all stroke (hazard ratio = 1.23, 95% CI: 1.47–4.04), ischemic stroke (hazard ratio = 1.31, 95% CI: 1.06–1.63), for coronary heart disease (hazard ratio = 1.29, 95% CI: 1.11–1.51), and all-cause mortality (hazard ratio = 1.16, 95% CI: 1.07–1.26) [33]. Some study reviewed scientific evidence and found similar results showing associations between routine consumption of non-nutritive sweeteners and increased health risk such as ischemic stroke, cardiovascular disease, type 2 diabetes and hypertension [32]. It also found biological mechanisms by which the artificial sweeteners may cause negative health effects. Therefore, the high-risk or susceptible population group should use these products with utmost caution. What is more, Thailand’s dietary guidelines should address the effects of artificial sweeteners and ASB consumption. People at risk, care givers and health professionals should also be amply informed about the potential risks of using them.

This study found that not smoking and having regular, vigorous physical activity were significantly associated with higher ASB consumption. This finding is in line with some studies, reporting that high ASB consumers are more likely to be non-smoker or former smoker [34] and people who have high physical activity [35] such as athletes and fitness enthusiasts. Participants with healthy lifestyle are usually concerned about harmful behaviors to health which include foods they eat - whether they could be harmful to their health. The food concern can regard the composition of their diet including sugar content of foods and beverages. Together, food marketing from beverage companies were created in response to regulatory actions on SSB by using various strategies, including ASB formulation and sales and their promotion as healthier alternatives to SSB [15]. Accordingly, ASB becomes an attractive option for cutting back on added sugar for maintaining a healthy diet among people pursuing a healthy lifestyle as a goal [36]. Therefore, developing specific communication strategies to educate the healthy-lifestyle group is needed to help them to make more informed healthy choices.

This study also found an association of certain sociodemographic factors with ASB consumption. For example, participants age 20–49 years were less likely to consume ASB compared with those age 50–64 years. It is possible that, as people age, those in a more senior age group are more likely to experience adverse health conditions at the same time. Diet is one of key modifiable risk factors for ill health [37]. Therefore, a management of an adverse health condition (e.g., diabetes) through diet management, including limiting sugar intake, is needed, and its effectiveness has been demonstrated elsewhere [38]. This may result in replacing diets of the older-age cohort with foods containing artificial sweeteners and other sugar-substituted foods with fewer or no calories, e.g., ASB. Moreover, people who live in Bangkok (capital city), the Central and Northeast regions were more likely to consume ASB [39] as compared to those in South region. This finding might be explained by food culture in each region. Compared to other regions, Southern Thai food is characterized by its spice and sharpness, with spicy and salty flavours [40]. Foods in other regions especially the Central and Bangkok have more neutral flavor - balancing sour, salty, spicy, bitter and sweet flavours. This difference may influence ASB consumption behaviours among people of different regions. Previous studies reported that people in these regions had greater consumption of SSB, particularly non-alcoholic carbonated and energy drinks, which contain a higher level of sugar than other types of SSB [41], compared with those residing in other regions [19]. With the growing concerns about the rise of obesity, diabetes, and other health problems, consumers in the two regions, therefore, may be more aware of this and, thus, may increasingly seek low- or no-calorie alternatives (such as ASB) to replace SSB. These findings point to the importance of improvement in food literacy of individuals to increase their ability to make their own healthy and safe food choices as well as for their family and community.

This study has some limitations. First, as the data collection was cross-sectional, causality cannot be inferred. However, this study was able to provide the most up-to-date, national overview of prevalence of ASB consumption in this group of Thai population, highlighting the key characteristics of ASB consumers and the associations between the socio-demographic, lifestyle, health factors and ASB consumption. Second, the authors acknowledge that the variable set used in this study did not include environmental and macro-level factors, and those can impact eating behaviours of individuals as well [42]. Future research is needed which analyses the interplay of multiple factors and their influence on ASB consumption. Moreover, the study population was mostly those in the working-age group, who live in an urban area, and had attained at least a Bachelor’s degree. The results of this study may not be applicable to the general population. However, the findings should be useful in the development of appropriate health policies and interventions in a specific population group – particularly the group with high ASB consumption, as those individuals should be government’s priority. This effort should be undertaken through policies and actions in food literacy and marketing control to improve the consumer profile, and create healthiness of the food environment which can lead to increasing access to healthy beverage products.

## Conclusions

The findings of this study suggest that being male, being age 20–49 years, living in Bangkok, the Central or the Northeast region, being a non-smoker, having adequate physical activity, having high BMI and having NCD were associated with high ASB consumption. This highlights the need for the Thai government to develop effective communication strategies that convey accurate, clear, and concise information to consumers regarding the health effects of ASB consumption in order for them to make their own healthy and safe food choices.

## Supporting information

S1 Table Socio-demographic and socio-economic characteristics of the HPBS sample. (DOCX)
