# Supplementary material for: Lifestyle, nutritional, and health influences on consumption of artificially-sweetened beverages in educated urban populations
Source: PLoS One. 2025 Apr 29;20(4):e0322553. doi: 10.1371/journal.pone.0322553 (PMC12040090; doi:10.1371/journal.pone.0322553)
Supplement: S1 Table — (DOCX) [file pone.0322553.s001.docx]

**S1 Table Socio-demographic and Socio-economic Characteristics of the HPBS Sample (N=62,891)**

| Variables | Number | Percent |
| --- | --- | --- |
| Sex |  |  |
| Male | 27,199 | 43.2 |
| Female | 35,692 | 56.8 |
| Age |  |  |
| 6-19 | 2,166 | 3.4 |
| 20-29 | 9,253 | 14.7 |
| 30-39 | 11,409 | 18.1 |
| 40-49 | 14,876 | 23.7 |
| 50-64 | 23,666 | 37.6 |
| 65+ | 1,521 | 2.4 |
| Education |  |  |
| Primary school or less | 27,121 | 43.1 |
| Secondary school | 25,169 | 35.7 |
| Bachelor’s degree or higher | 13,339 | 21.2 |
| Marital status |  |  |
| Single | 15,044 | 23.9 |
| Married | 39,538 | 62.9 |
| Widowed, divorced, separated | 8,309 | 13.2 |
| Place of residence | | |
| Urban | 30,271 | 48.1 |
| Rural | 32,620 | 51.9 |
| Area |  |  |
| Bangkok | 8,649 | 13.8 |
| Central | 20,028 | 31.8 |
| North | 10,629 | 16.9 |
| Northeast | 15,634 | 24.8 |
| South | 7,950 | 12.6 |
| Poverty |  |  |
| Poor | 11,084 | 17.6 |
| Not Poor | 51,807 | 82.4 |
| Smoke |  |  |
| No | 51,679 | 82.2 |
| Yes | 11,212 | 17.8 |
| Drink alcohol |  |  |
| No | 53,902 | 85.7 |
| Yes | 8,989 | 14.3 |
| Physical activity |  |  |
| No | 35,911 | 57.1 |
| Yes | 26,979 | 42.9 |
| Disease |  |  |
| No | 49,584 | 78.8 |
| Yes | 13,306 | 21.2 |
| BMI |  |  |
| Underweight | 3,639 | 5.8 |
| Normal weight | 27,602 | 43.9 |
| Overweight | 12,415 | 19.7 |
| Obesity Class 1 | 15,063 | 24.0 |
| Obesity Class 2 | 4,172 | 6.6 |
| ASB consumption (in the past week) |  |  |
| No | 60,786 | 96.7 |
| Yes | 2,105 | 3.3 |
| Total | **62,891** | **100.0** |
